# *In vitro* comparison of antimicrobial effectiveness of QMix and other final irrigants in human root canals

**DOI:** 10.1038/srep17823

**Published:** 2015-12-03

**Authors:** Ying Liu, Lili Guo, Yuqin Li, Xiangjun Guo, Bin Wang, Ligeng Wu

**Affiliations:** 1 Department of Endodontics, School of Stomatology, Tianjin Medical University, Tianjin, China; 2 Department of Stomatology, The First Affiliated Hospital of Henan University of TCM, Henan, China; 3 Department of Stomatology, Affiliated Hospital of Guiyang Medical College, Guizhou, China; 4 Department of Endodontics, School of Stomatology, Tianjin Medical University, Tianjin, China; 5 Department of Endodontics, School of Stomatology, Tianjin Medical University, Tianjin, China

## Abstract

Final root canal irrigation stands as an effective strategy for eliminating the dentin infection. This study aimed to investigate and compare the antibacterial efficacy of QMix and other four final irrigation regimens in reducing *Enterococcus faecalis* within human root canals. Single-canal human teeth contaminated with *E. faecalis* for 4 weeks were prepared chemomechanically with sodium hypochlorite (NaOCl). Then, the teeth were randomly assigned into six groups according to the final irrigation protocols: (1) EDTA/NaOCl, 17% EDTA followed by 5.25% NaOCl; (2) EDTA/chlorhexidine (CHX), 17% EDTA followed by 2% CHX; (3) EDTA/cetrimide (CTR), 17% EDTA followed by 2% CTR; (4) MTAD; (5) QMix; and (6) control, 0.9% saline. Bacterial samples collected before instrumentation and after final irrigation were cultured and the colony-forming units (CFUs) were counted. The CFUs in the QMix, EDTA/CHX, and EDTA/CTR groups were significantly lower than those in the EDTA/NaOCl group. No significant differences were observed between the QMix, EDTA/CHX, and EDTA/CTR groups. MTAD showed weaker ability than QMix and EDTA/CHX to eliminate *E. faecalis,* but it caused a greater reduction in CFU than EDTA/NaOCl. Hence, the antimicrobial activity of QMix was comparable to that of EDTA/CHX and EDTA/CTR and more effective than that of EDTA/NaOCl against intracanal *E. faecalis*.

Bacteria and their metabolic byproducts are the main etiological agents that cause endodontic diseases and periapical lesions. However, eliminating microorganisms from the root canal system after root canal preparation is difficult because of its anatomical complexity^1^. Therefore, root canal irrigation has an indispensable role in reducing microbes in areas that are inaccessible to instruments^2^.

*Enterococcus faecalis* is the major pathogen isolated from failed root canals and persistent periapical lesion^3^,^4^ and is found in 24%–77% of root–filled teeth with periradicular lesions^5^,^6^,^7^,^8^,^9^. Complete elimination of *E. faecalis* from the root canal system is difficult because of its ability to penetrate into the dentinal tubules and its tolerance to harsh environments such as nutrient deficiency^10^, high salt concentrations^11^, extreme alkaline environment (pH = 9.6)^12^, and antimicrobial agents^13^. In recent years, final irrigation techniques have emerged to enhance root canal cleaning by providing antimicrobial activity not only during application but also maintaining activity after treatment^14^. Furthermore, the sequential use of a chelating agent in combination with antiseptics or antibiotics is recommended as the final irrigation protocol.

QMix is a novel one–step irrigating solution that contains a mixture of bisbiguanide antimicrobial agent, calcium–chelating agent, saline, and surfactant. QMix is reportedly as effective as 17% ethylenediaminetetraacetic (EDTA) in reducing the smear layer^15^,^16^. QMix has also been proven to have effective antimicrobial activity for the disinfection of hydroxyapatite discs infected with *E. faecalis*^16^. However, to date, little information is available on the antimicrobial ability of QMix in human teeth infected with *E. faecalis*.

Numerous studies have reported that chlorhexidine (CHX) decreases *E. faecalis* populations in root canals^17^,^18^ and has the property of substantivity^19^. However, sequential use of EDTA with CHX in the final irrigation of human teeth infected with *E. faecalis* has not been previously investigated. Cetrimide (CTR) has also been shown to possess effective antimicrobial activity against an *E. faecalis* biofilm and reduce biofilm mechanical stability^20^,^21^. Ferrer–Luque *et al.*^22^ reported the antimicrobial activity of EDTA combined with CTR as a final irrigating solution for root canals infected with *E. faecalis*; however, the results were restricted to residual antimicrobial activity. The most commonly used final irrigation regimens include the use of EDTA, followed by sodium hypochlorite (NaOCl), which exhibits the capacity to remove the smear layer and eliminate the biofilm^23^,^24^. MTAD is known to remove the smear layer, disinfect contaminated root canals, and eliminate *E. faecalis*^25^,^26^,^27^. However, the antimicrobial efficacy of EDTA/NaOCl versus MTAD is controversial^27^,^28^.

In this study, we evaluated the antimicrobial activity of QMix, MTAD, 17% EDTA/5.25% NaOCl, 17% EDTA/2% CHX, and 17% EDTA/0.2% CTR as the final irrigation regimens against *E. faecalis* biofilms within human root canals.

We hypothesized that the novel final irrigation regimens, QMix, 17% EDTA/2% CHX and 17% EDTA/0.2% CTR, would have effective antimicrobial activity in human teeth infected with *E. faecalis*.

## Results

### Observation in Scanning Electron Microscopy (SEM)

**Figure 1:**
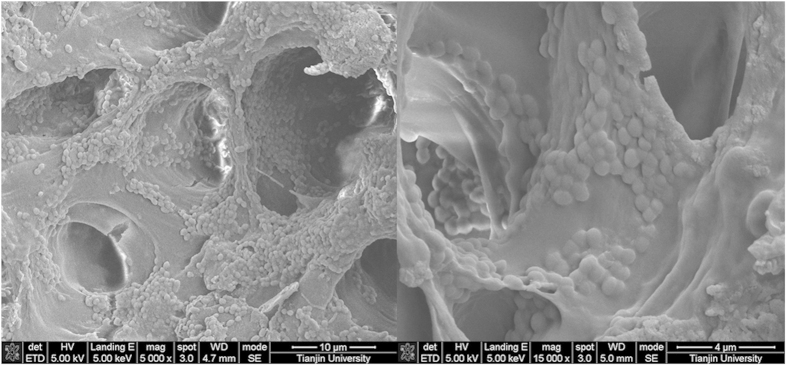
Scanning electron micrograph showing colonization of the root canal walls by *E. faecalis* (left: 5000× , right: 15000× ).

The SEM examination revealed that the root canal walls were colonized by dense *E. faecalis* biofilm–like structures. At high magnification (10,000×), *E. faecalis* was clearly observed singly, in pairs, or in short chains invading into dentinal tubules (Fig. 1).

### Microbiological Analysis

**Figure 2:**
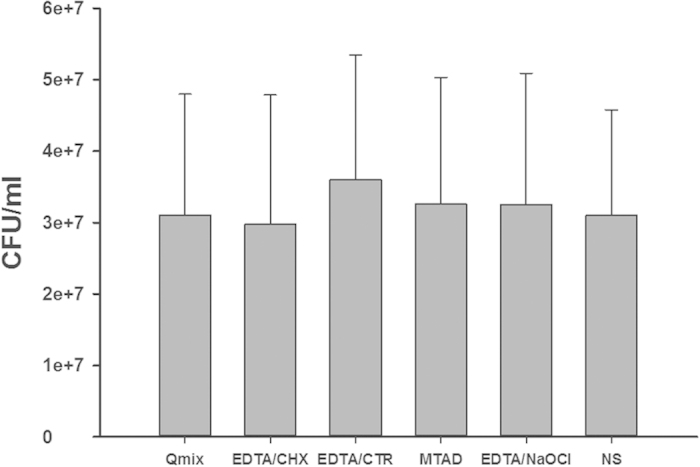
Mean and standard deviation of *E. faecalis* CFU counts for all groups before chemomechanical preparation (S1). No statistical differences were observed between the different groups (P > 0.05).

**Figure 3:**
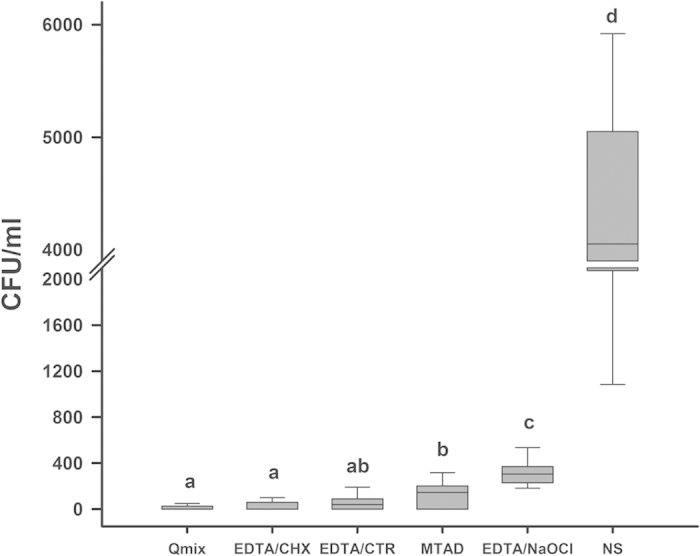
Median and interquartile range of CFU counts for all groups after final irrigation of various final irrigation protocols (S2). The superscript without same letters indicates statistical significance.

Figure 2 shows the mean and standard deviation of CFU counts for all groups before chemomechanical preparation (S1). No significant differences were observed between these groups (P > 0.05). Figure 3 shows the median and interquartile range of CFU counts for all groups after the final irrigation by the various final irrigation protocols (S2). The results showed that all experimental groups were significantly more effective than the control group in reducing *E. faecalis* (P = 0.000). EDTA/CHX, EDTA/CTR, or QMix exhibited the greatest antimicrobial activity. No significant differences were observed between these three groups (P = 0.266). MTAD was more effective than EDTA/NaOCl in reducing the bacterial counts (P = 0.003).

The frequency of negative cultures in S2 was as follows: 0 of 10 cultures (group A), 6 of 10 cultures (group B), 4 of 10 cultures (group C), 3 of 10 cultures (group D), 6 of 10 cultures (group E), and 0 of 10 cultures (control), as shown in Table 1. No statistical differences existed between the experimental groups B, C, D and E (P = 0.538).

## Discussion

The pathogenicity of *E. faecalis* in causing persistent periapical lesions and root canal failure is well recognized. In the present study, an E. faecalis–infected root canal biofilm model was established to investigate the bacterial removal efficiency of different final irrigation regimens in human root canals. Root canals were infected for 4 weeks to ensure the maturation of the biofilm. The antimicrobial abilities of QMix and the five other final irrigation protocols were compared.

To date, a thorough eradication of *E. faecalis* biofilm in root canals cannot be achieved with a single irrigating solution. The combined use of different irrigants is therefore necessary to enhance antimicrobial effectiveness. In this study, the final irrigation regimens contained an antimicrobial agent and a smear layer removal agent. MTAD and QMix are multi-component mixed solutions for smear layer removal and disinfection in one step. In the other three experimental protocols, EDTA was used in combination with another antimicrobial agent because of its efficiency in smear layer removal.

The present *in vitro* study showed that intracanal *E. faecalis* populations obtained before root canal preparation were vast, with quantities up to 10^7^ CFU/mL. After chemomechanical preparation and final irrigation, the CFU data decreased significantly for all groups. Furthermore, the five experimental groups showed greater antimicrobial activity than the control group. In a word, both the chemomechanical preparation and final irrigation reduced the intracanal bacteria numbers with the promise of optimizing root canal disinfection.

The irrigation protocols using EDTA/CHX, EDTA/CTR, and QMix obtained the best and comparable results for antibacterial efficiency against intracanal *E. faecalis*, whereas EDTA/NaOCl showed the least antimicrobial capability and MTAD presented intermediate effects. Teeth treated with QMix showed the lowest number of bacteria and the greatest frequency of negative cultures after the final irrigation.

Stojicic *et al.*^16^ reported that QMix effectively killed *E. faecalis* biofilms grown on collagen-coated hydroxyapatite discs *in vitro*, and was superior to CHX and MTAD. The findings of our study did not strictly agree with these results, which may be attributed to the different study models. The *E. faecalis* biofilm established by Stojicic *et al.* was on collagen-coated hydroxyapatite discs rather than on human root canals. However, the excellent antimicrobial activity of QMix has been further demonstrated in this study. The advantages of QMix may be attributed to its various effective components, which include EDTA, CHX, and a detergent (surface active agent). The addition of a surface active agent contributes to lowering the surface tension of solutions, which can increase wettability for better penetration into the dentinal tubules. Moreover, QMix has been shown to be as effective as EDTA in removing the smear layer and is more biocompatible than many other irrigants^29^. Therefore, our results demonstrate that QMix is a promising irrigating solution for disinfecting infected root canals. However, further clinical studies are needed.

The combination of EDTA with CHX as a final irrigating protocol to reduce intracanal *E. faecalis* has not previously been explored. Our study demonstrated that EDTA/CHX was significantly more efficient than MTAD or EDTA/ NaOCl as the final irrigation regimen. Our results also demonstrated the high antimicrobial efficiency of EDTA/CTR against *E. faecalis*, which is in agreement with the results of Ferrer–Luque *et al.*^22^ However, our study examined the effects of irrigants in root canals immediately after the final rinse while Ferrer–Luque assessed the residual antimicrobial activity by observing sample turbidity daily.

Previous studies have attempted to assess the antimicrobial efficacy of MTAD versus EDTA/NaOCl as the final irrigation regimens. However, the results of these studies have been conflicting. Shabahang and Torabinejad^27^ reported that a combination of 1.3% NaOCl with MTAD as the final irrigant was significantly more effective against *E. faecalis* than EDTA/NaOCl. By contrast, Dunavant *et al.* demonstrated that 6% and 1% NaOCl were significantly more efficient in eliminating intracanal bacteria than MTAD^28^. In addition, no difference in the antimicrobial efficacy of NaOCl/EDTA versus MTAD was demonstrated by Kho and Baumgartner in the apical 5 mm of roots contaminated with *E. faecalis*^30^ In this study, the results were in agreement with those obtained by Shabahang and confirmed the higher efficiency of MTAD in reducing the *E. faecalis* biofilm.

## Conclusion

The final irrigation regimens were highly significant in eliminating intracanal *E. faecalis* biofilms after instrumentation. The antimicrobial efficacy of Qmix, 17% EDTA/2% CHX, and 17% EDTA/0.2% CTR were effective, comparable, and reliable. Our hypothesis could therefore be accepted. QMix, a novel single irrigant, demonstrated potential as a final irrigant for root canals due to its advantages of easy manipulation and efficient antimicrobial properties.

## Materials and Methods

### Irrigating Solutions

Five root canal irrigants were used in the present study: EDTA (Meta, Korea), MTAD (Dentsply Tulsa Dental, OK, USA), QMiX (Dentsply, Tulsa Dental, OK, USA), CTR (Sigma–Aldrich, Shanghai, China), and CHX (Sigma–Aldrich, Shanghai, China).

### Specimen Preparation and Contamination

The study protocol was approved by the Ethics Committee of the Tianjin Medical University (Written ethics review sheet number: TMUhME2013041). This study was conducted in full accordance with the World Medical Association Declaration of Helsinki. All study participants provided informed consent in written form, and the consent procedure was approved by the ethics committee. Sixty–seven extracted human single–rooted maxillary anterior teeth were selected. The teeth were curetted to remove soft tissue, debris, and calculus from the root surface. Each tooth was decoronated to obtain a 12–mm root from the anatomical apex followed by the removal of the pulp. To obtain a closed root canal system that mimicked clinical *in vivo* scenarios and was easy to handle, a customized model was fabricated for each tooth, based on the protocol by Johal *et al.*^31^. In brief, each tooth was immersed into a 1.5-mL polypropylene Eppendorf tube filled with silicone rubber. The teeth and models were then autoclaved at 121 °C, 1.5 MPa for 30 minutes. After sterilization, 5 teeth were randomly selected to be cultured in brain-heart infusion (BHI) at 37 °C as the negative controls.

*E. faecalis* (ATCC 29212) was cultured overnight in BHI broth to obtain a concentration of 1 × 10^7^ cells/mL. In accordance with the protocol described by Baca *et al.*^32^, *E. faecalis* was then allowed to incubate for 4 weeks under aerobic conditions at 37 °C, with the medium replaced by fresh culture medium every other day. The cultures were random sampled to check the purity of cultures by Gram stain and colony morphology when replacing the medium. Two random samples were subjected to scanning electron microscopy (SEM) to verify the presence of the *E. faecalis* biofilm. The method of lottery was applied to select two samples from the 62 samples. SEM photographs were obtained from three randomly selected locations at different sites (apical third, middle third and coronal third).

The *E. faecalis*–contaminated root canals were established after 4 weeks. To determine the working length, a size10 K–file (Dentsply Maillefer, Shanghai, China) was inserted and the apex of each tooth was sealed using cyanoacrylate. Each tooth was thereafter embedded into its respective customized model. The cyanoacrylate sealed the interface between the outer tooth surface and the impression material. The root canals were prepared using the rotary ProTaper system (Dentsply Maillefer, Shanghai, China) to F3 in a crown–down manner. During preparation, each canal was irrigated with 1 ml of 5.25% NaOCl after each file.

After instrumentation, the roots were randomly assigned into six groups (n = 10 teeth per group) according to the final irrigation regimens: (1) EDTA/NaOCl, 17% EDTA followed by 5.25% NaOCl (group A); (2) EDTA/CHX, 17% EDTA followed by 2% CHX (group B); (3) EDTA/CTR, 17% EDTA followed by 2% CTR (group C); (4) MTAD (group D); (5) QMix (group E); and (6) saline (control). The amount of each irrigant was 5 mL. All the irrigating solutions were administered in accordance with the manufacturer’s instructions by using a 30–gauge side-vented needle (Dentsply Maillefer, Shanghai, China) inserted into each canal 1 mm short of the working length, and with 1 mL of saline used between irrigants to avoid interaction.

### Sampling Procedures

Samples were taken before (S1) the chemomechanical procedures and after (S2) the final root canal irrigation as follows: first, root canals were dried with sterile paper points and refilled with sterile BHI broth. Next, a 15^#^ K–file was then inserted into the root canal up to the working length and circumferentially filed for 20 seconds. Three consecutive sterile paper points were then placed into the canal to absorb the root canal contents, which were then transferred to 5 mL of BHI broth followed by 10–fold serial dilutions in saline. Finally, aliquots (100 μL) were spread onto BHI agar and incubated at 37 °C for 48 hours, after which the number of colony-forming units (CFUs) was counted.

### Statistical Analysis

The S1 data of all groups (experimental and control) followed the standard normal distribution, therefore a one-way analysis of variance (ANOVA) was applied to compare the initial infection between the groups. For inter–group comparison, the Kruskal–Wallis test was used for comparative analysis of the S2 data. The Fisher’s exact test was performed to compare the occurrence of negative cultures in the S2 data. The statistical significance level was established at P < 0.05. Analyses were performed via SPSS 17.0 software (SPSS, Chicago, IL).

## Additional Information

**How to cite this article**: Liu, Y. *et al.* *In vitro* comparison of antimicrobial effectiveness of QMiX and other final irrigants in human root canals. *Sci. Rep.* **5**, 17823; doi: 10.1038/srep17823 (2015).

## Figures and Tables

**Table 1:** Frequency of Negative Cultures of Various Final Irrigation Protocols.

	A	B	C	D	E	Control
Negative cultures	0^a^	6^b^	4^ab^	3^ab^	6^b^	0^a^

A: EDTA/NaOCl; B: EDTA/CHX; C: EDTA/CTR; D: MTAD; E: QMiX. Groups with different superscript letters have a statistically significant difference.
